# 106. Pandemic Pinch: The Impact of COVID Response on Antimicrobial Stewardship Program (ASP) Resource Allocation

**DOI:** 10.1093/ofid/ofab466.308

**Published:** 2021-12-04

**Authors:** Elizabeth Dodds Ashley, April Dyer, Travis M Jones, Melissa D Johnson, Angelina Davis, Katherine R Foy, Alicia Nelson, Sonali D Advani, Sonali D Advani, Andrea Cromer, Danielle Doughman, Ibukunoluwa Akinboyo, Emily Sickbert-Bennett, Rebekah W Moehring, Deverick J Anderson, Steven S Spires

**Affiliations:** 1 Duke Center for Antimicrobial Stewardship and Infection Prevention, Durham, NC; 2 Duke University School of Medicine, Durham, North Carolina; 3 Duke University, Durham, NC; 4 Duke University School of Medicine, Duke Infection Control Outreach Network, Durham, NC; 5 Duke Infection Control Outreach Network (DICON), Inman, South Carolina; 6 University of North Carolina Medical Center, Chapel Hill, North Carolina; 7 UNC Health Care, Chapel Hill, NC

## Abstract

**Background:**

The COVID-19 pandemic placed a strain on inpatient clinical and hospital programs due to increased patient volume and rapidly evolving data on best COVID-19 management strategies. However, the impact of the pandemic on ASPs has not been well described.

**Methods:**

We performed a cross-sectional electronic survey of stewardship pharmacy and physician leaders in 37 hospitals within the Duke Antimicrobial Stewardship Outreach Network (DASON) (community) and Duke/UNC Health systems (academic) in April-May 2021. The survey included 60 questions related to staffing changes, use of COVID-targeted therapies, related restrictions, and medication shortages.

**Results:**

Twenty-seven facilities responded (response rate of 73%). Pharmacy personnel was reduced in 17 (63%) facilities by an average of 16%. Impacted pharmacy personnel included the stewardship lead in 15/17 (88.2%) hospitals. Converting to remote work was rare and only reported in academic institutions (n=2, 7.4%). ASP personnel were reassigned to non-stewardship duties in 12 (44%) hospitals with only half returning to routine ASP work as of May 2021. Respondents estimated that 62% of routine ASP activities were diverted during the time of the pandemic. Non-traditional, pandemic-related ASP activities included managing multiple drug shortages, of which ventilator support medications (91%) were most common affecting patient care at 52% of facilities. Steroid and hydroxychloroquine shortages were less frequent (44% and 22%, respectively). Despite staff reductions, pharmacists often served as primary contact for remdesivir approvals either using a criteria-based checklist at dispensing or as part of a dedicated phone approval team (Figure). Most (77%) hospitals used a criteria-based pharmacist review strategy after remdesivir FDA approval. Restriction processes for other COVID-19 therapies such as tocilizumab, hydroxychloroquine, and ivermectin were reported in 64% of hospitals.

Remdesivir Allocation Strategy

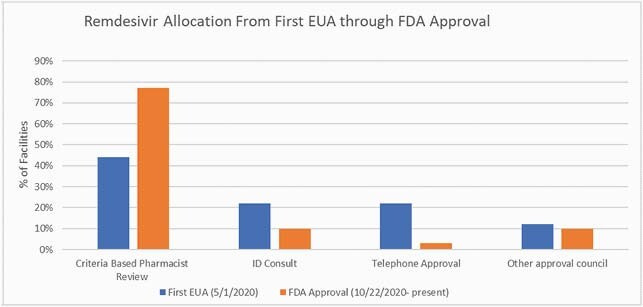

Proportion of facilities implementing specific remdesivir allocation strategies from the time of the first US Food and Drug Administration (FDA) Emergency Use Authorization (EUA) through FDA approval

**Conclusion:**

Pandemic response diverted routine ASP work and has not yet returned to baseline. Despite the reduction in pharmacy personnel due to the pandemic, the ASP pharmacy lead took on a novel and critical stewardship role throughout the pandemic exemplified by their involvement in novel treatment allocation for COVID patients.

**Disclosures:**

**Melissa D. Johnson, PharmD, MHS**, **Astellas** (Consultant, Grant/Research Support)**Charles River Laboratories** (Grant/Research Support)**Cidara** (Consultant)**Merck & Co** (Consultant, Research Grant or Support)**Paratek** (Consultant)**Pfizer** (Consultant)**Scynexis** (Scientific Research Study Investigator)**Theratechnologies** (Consultant)**UpToDate** (Other Financial or Material Support, Author Royalties) **Sonali D. Advani, MBBS, MPH**, Nothing to disclose **Rebekah W. Moehring, MD, MPH**, **UpToDate, Inc.** (Other Financial or Material Support, Author Royalties)

